# Competence-based education misses the essence of the medical profession

**DOI:** 10.1007/s40037-015-0233-5

**Published:** 2015-11-09

**Authors:** M.J.M.H. (Kiki) Lombarts

**Affiliations:** Professional Performance Research Group, Center for Evidence-Based Education, Academic Medical Center, University of Amsterdam, Amsterdam, The Netherlands

## Essentials

- Competence-based education (CBE) models ought to be critically reviewed in terms of their contribution to training for high-performing, empathic physicians.
- Physician disengagement, mediocrity in training outcome, and deprofessionalization, may be unintended consequences of the introduction of CBE models in the medical profession. None are of service to patients.
- Complex professional realities can never be appreciated by the behaviourist CBE model, nor can it be captured in checklists, assessments and technical forms.
- The effectiveness or superiority of CBE models, should be evidenced in terms of improved patient outcomes.

It is not easy to take a stance against what seems to be widely accepted: competence-based education [1, 2]. Given its ultimate aim of providing excellent patient care, however, a pause for reflection may be necessary. In this debate I will regard competence-based education models simply as those models in which the unit of education and training (as well as planning and assessment) is a competency, and a set of competencies are intended to capture physician competence.

Patients expect and deserve high-performing, empathic physicians and must be able to trust that academic training centres serve as the source for creating this prototype of physician. I will argue that competence-based education frameworks may undermine training for true professionalism and highlight how their use challenges the professional status of the medical profession. Hereto, I will bring forward two theoretical arguments, referring to the literature on expert performance and the sociology of professions. In short, there are valid reasons to state that the current competence-based approaches (i) elicit educating for mediocrity instead of excellence and (ii) facilitate the process of *de*professionalization of the medical profession; both being of disservice to the quality of patient care. First, however, I will put the spotlight on potentially the most important argument, because it is empirically based: the competency frameworks’ unintended effects of alienating and disengaging physicians from what it means to be a doctor. Unsurprisingly, many physicians (both faculty and trainees) are not at ease with their professional realities—consisting of interacting complex social phenomena of teaching, learning and taking care of patients—being narrowed down to distinct competencies and measurable behaviours and skills. They may perceive the behaviourist view underlying the competence-based model—to operationalize academic and professional knowledge and skills in prescriptions for behaviour—as an impoverished view of professionalism [3] feeling the inadequacy of the model in capturing the core of professional practice. Physicians experience and oppose, for example, the bureaucratic formats through which competence-based models have found their way into practice. They report feeling overwhelmed by the number of imposed checklists, online assessments and administrative requirements that seem to underappreciate the complexity of clinical and teaching practice [1]. The instrumental approach of ‘signing off’ the professional development of students or residents seems to demoralize both faculty and trainees [4]. It pushes them to hide behind schemes, rules and regulations, instead of encouraging them to assume their professional responsibilities and invest their enthusiasm, expertise and skills in training future colleagues and delivering patient care. In the transition to competence-based education it has proven difficult to maintain the holistic views on professionalism and professional development that reality merits.

## High-performing physicians and health care systems

Ground-breaking research into the phenomenon of expert performance and expertise has been done by the Swedish psychologist Ericsson [5, 6]. Based on decades of research we now know that ‘excellent performers are made, not born’. Achieving excellent performance levels requires exposure and experience (clocking up flying hours), dedication to deliberate practice and a drive for improvement. More specifically, clinical excellence derives from intrinsic motivation, passion for patient care, humility and exercising reflection, scholarship and being adaptive [7, 8]. Importantly, excellence is about integrating all these qualities. Reviewing the current competency frameworks, and their implementation in teaching and training, one wonders if they offer the best impetus and focus for creating the much wanted high-performing physicians. I worry that primarily focusing on the prescribed behaviours may prevent or distract clinical teachers and their trainees from paying attention to training-for-excellence. Symptoms of misalignment with the key elements of excellence are, for example, isolated courses on separate competencies and the declining empathy levels amongst young doctors [9], denying the integrative, motivational and reflective aspects of excellence, respectively. I worry that the competence-based models prime trainees to professionally comply with the predefined behaviours, but do not train them to become true medical doctors. Whitehead et al. have underlined how behavioural models limit or even deny relational and situational factors, values and the role of the personhood [10]. None of these can be missed in striving for excellence. Competence-based education may not lead to the best possible performance; by focusing on behaviours and skills we may well be educating for mediocrity, not excellence.

## The professional status of the medical profession

My last critique on competence-based models comes from my understanding of the nature of the medical profession and its relationship with society. This relationship has been referred to as a ‘social contract’, meaning that society grants the medical profession the privilege of self-regulation, as long as physicians make patients’ interests their first priority, demonstrate integrity, morality and their commitment to excellence [11]. The privilege of self-regulation boils down to the profession defining and monitoring its own quality standards, educating and training future peers, and regulating entrance to and removal from the profession. In theories on the professions and professionalism, doctors deserve this self-regulating authority based on their unique body of knowledge, the complexity of professional practice and their dedication to serving patients’ interests, as laid down in the Hippocratic oath. If executed well, the self-regulating role of the medical profession may best assure the quality of patient care, and protect patients from too much influence of governmental and market powers (commercialism) [12]. If the medical profession has to give in to its privileges, this can be seen as a process of *de*professionalization. Instead of defining and controlling patient care, doctors than, in ultimo, become the executers of care which may be designed or prescribed by layman. We have witnessed this deprofessionalization process over the past decades, when third-party payers, hospital managers, quality experts and patient safety professionals gained substantial grounds in the medical profession. Consequently, physicians have been confronted with redesigned patient care processes, told to comply with new quality and safety standards and protocols, and made to measure and register their interactions in detail. Indeed, they have lost some self-steering power. In this line of thinking, educationalists may be seen as the next group of experts to enter the medical profession. By pushing competence models—a new *logic*—and life-long competence-based education—indeed a new *practice*—the medical profession’s self-regulating practices and privileges may slowly be further trimmed down. Competence-based models thus fuel the process of *de*professionalization. Before positioning non-doctors to take control over another part of medical practice as we know it today, society deserves to first see the evidence of the superior idea of competence-based education. If I were a physician I would want to see this evidence in terms of improved patient outcomes.

## To conclude

It is primarily up to the medical community to protect its status as a profession, including the reward of self-regulation. Competence schemes may be useful for promoting the development of specific skills and behaviours, but are not sufficient to educate or train excellent performing physicians nor to protect society’s trust in the medical profession. Many physicians have experienced the technocratic consequences of competence schemes. Physicians may want to return from a not-too-critical adoption of competence frameworks to creative and constructive dialogues about the core of what it is to be a professional, and how to best build a doctor who is fit for modern practice [13]. That indeed would be an act of professional self-empowerment. One that would facilitate the process of professionalization, strengthen individual physicians, unify the medical profession and build trust with society.
